# What we know and don’t know about the antenatal care service utilization in Ethiopia: A scoping review of the literature

**DOI:** 10.1371/journal.pone.0321882

**Published:** 2025-05-30

**Authors:** Amanuel Yoseph, Kibru Kifle, Yohannes Seifu Berego, Mehretu Belayneh, Alemu Tamiso, G. Mutwiri, Francisco Guillen-Grima

**Affiliations:** 1 School of Public Health, College of Medicine and Health Sciences, Hawassa University, Hawassa, Ethiopia; 2 School of Medicine, College of Medicine and Health Sciences, Hawassa University, Hawassa, Ethiopia; 3 Department of Environmental Health, College of Medicine and Health Sciences, Hawassa University, Hawassa, Ethiopia; 4 School of Public Health, University of Saskatchewan, Saskatoon, Saskatchewan, Canada; 5 Department of Health Sciences, Public University of Navarra, Pamplona, Spain; 6 Group of Clinical Epidemiology, Area of Epidemiology and Public Health, Healthcare Research Institute of Navarre (IdiSNA), Pamplona, Spain; Nazarbayev University School of Medicine, KAZAKHSTAN

## Abstract

**Introduction:**

In Ethiopia, there has been considerable recent investment and prioritization in the maternal health program. However, coverage rates have been low and stagnant for a long time, indicating the existence of systemic utilization barriers. Therefore, it is fundamental to synthesize the current body of knowledge to successfully address these problems and enhance program effectiveness to increase antenatal care (ANC) uptake.

**Methods:**

We conducted a scoping review of the literature. Using various combinations of search strategies, we searched Pubmed/Medline, WHO Library, ScienceDirect, Cochrane Library, Google Scholar, and Google for this review. Preferred Reporting Items for Systematic Reviews and Meta-Analyses Extension for Scoping Reviews (PRISMA-ScR) were used to conduct the review. We included studies that used any study design, data collection, and analysis methods related to antenatal care utilization.

**Results:**

A total of 76 studies, national surveys, and estimates were included in this review. The analysis revealed that ANC utilization coverage varied considerably by region, from 27% in Somali to 90.6% in the Oromia region, with significant disparities in socioeconomic status, access to healthcare, and vaccination knowledge. Ten priority research areas covering various aspects of the national ANC services were identified through a comprehensive review of the existing body of knowledge led by experts using the Delphi method.

**Conclusion:**

The barriers to recommended ANC service utilization differ depending on the context, suggesting that evidence-based, locally customized interventions must be developed and implemented. This review also identified evidence gaps, focusing on health system-related utilization barriers at the lower level, and identified additional research priorities in Ethiopia’s ANC service. The first step in developing and executing targeted program approaches could be identifying coverage of ANC services utilization among those with disadvantages.

## Introduction

A woman carries and conceives a growing fetus inside her womb during pregnancy, which is a normal physiological process. Several steps are involved, all of which are essential to delivering a healthy child [1,2]. As a result, there is a need to invest in maternal health care to experience a positive pregnancy outcome during the critical period. The investment includes financing or subsidizing the cost of healthcare, supporting the use of effective contraceptives, promoting ANC use, educating women about their health, and ensuring follow-up on the health of women and their newborns [3].

Considering this, several global initiatives have been launched to improve maternal health. These initiatives include the Nairobi Safe Motherhood Conference (1987) [4], the Cairo International Conference on Population and Development (ICPD) (1994) [5], the Millennium Development Goals (MDGs) conference (2000) [6], and the most recent global sustainable development goals conference (2016), which focused on reducing maternal illness and death and improving child health [7]. Besides, the World Health Organization (WHO) strives to improve maternal health by providing evidence-based clinical and programmatic guidance, improving research evidence, establishing global standards and criteria, and providing member nations with technical support in developing and implementing successful policies and programs [8].

The Ethiopian government has been working hard to improve maternal health per WHO programs. Among these measures is the development of a robust and comprehensive 20-year Health Sector Development Program (HSDP), which is broken down into four phases. The main goal of each phase is to improve the utilization of maternal health services through a five-year investment plan [9]. Health Extension Programs (HEPs), which were launched in 2003 with a particular emphasis on preventive health service provision and health education at the community level, are thought to be the most important interventions that can help lower the rate of maternal illness and death [10]. Six years after its launch, a new innovative approach, the Women’s Development Army (WDA), was linked with HEP in 2010. The WDA is a structural organization involving one to five linkages and six households in the same neighborhood. The community settings aim to promote early identification and link pregnant women to nearby health facilities to increase the proportion of ANC use [11]. A national reproductive strategy (2016–2020) designed to enable communities, families, and women to recognize pregnancy-related obstetric danger signs (ODS) and foster safe motherhood [12]. A growth and transformation plan (GTP) that includes a pregnant women forum, which is essential for teaching about ODS and the birth preparedness and complication redness (BPCR) plan, training and deployment of health care providers (HCPs), especially midwives, in rural areas to provide community-based maternal health services, and the expansion of health facilities [13]. Despite all international and national efforts, maternal mortality remains unacceptably high, with one woman dying globally every two minutes from direct and indirect causes due to low maternal health service utilization [14].

ANC continues to be an important intervention for improving maternal and neonatal health outcomes [14,15]. It allows pregnant women and healthcare providers to talk about proper nutrition, identifying ODS, and developing a childbirth plan [16], preventive care, like the provision of folic acid or ferrous sulfate tablets and tetanus toxoid vaccination [15]. The WHO recommends at least 8 ANC visits for women with a normal pregnancy to provide users with a more woman-centered and positive experience, particularly in countries with limited resources [15].

However, globally only 57% of women utilized at least four ANC services compared to 90% of women who utilized at least one; in Africa, 53% and 49% of women, respectively, received at least one and four visits [17]. Similarly, only 43% of pregnant women utilized four or more ANC visits in Ethiopia [18]. The proportion of ANC utilization differs by regions, sub-regions, and districts in Ethiopia, ranging from 12 to 94.8% [18]. This disparity is one of the main obstacles preventing Ethiopia from achieving maternal and newborn mortality statistics in line with SDG3 [7].

According to the 2019 Mini Ethiopian Demographic and Health Survey (EDHS), at least one ANC service utilization increased from 28% in 2005 to 74% in 2019 [18]. Nevertheless, little achievement has been made in closing the regional state gap, and Ethiopia’s urban-rural disparity remains high [18–21]. Also, about one-third of women who use ANC services do not receive all services packages during their follow-up [18]. Further, socio-cultural beliefs, such as considering pregnancy as water or blood in the womb before four months of gestation, hampered the timely initiation of ANC services [22].

The Ethiopian government has developed several strategies to overcome the low utilization of ANC and the disparity between regions and urban and rural settings. However, implementing the plans and strategies is difficult due to several barriers, such as access, service delivery approaches, health facility and health professional shortages, community engagement, socio-cultural factors, and service quality [18,22,23]. There is also a lack of a comprehensive understanding of ANC service barriers, determinants, and implementation bottlenecks that impede adequate ANC utilization coverage and quality [18,24]. Given the circumstances, little is known about Ethiopia’s ANC service implementation issues or their underlying determinants, barriers, and facilitators. Thus, a comprehensive literature review was done to inform implementers, managers, and policy/decision makers about ANC utilization and identify research gaps on the epidemiology of ANC utilization in Ethiopia.

## Rationale of the scoping review

Epidemiological evidence is essential for creating effective intervention strategies and addressing knowledge gaps. To close this knowledge gap, the scoping review of the literature will help to clarify the present level of understanding regarding the execution of the ANC service. Additionally, this scoping review prioritized future research areas related to Ethiopia’s ANC service utilization and found significant implementation gaps. The findings of this review will help program managers, decision-makers, and implementers find effective promotion strategies to improve ANC and meet the SDGs. The findings can inform evidence-based decision-making for Ethiopian women facing challenges throughout their pregnancy care journey. This study provides valuable insights for maternal health advocates in Ethiopia, identifying determinants, barriers, and facilitators of ANC. Furthermore, it offers evidence-based recommendations to optimize resource utilization. Moreover, it serves as a source of information for future research.

## Objectives of the review

### General objective

The general objective of this scoping review of the literature was to investigate the current level of knowledge regarding the barriers to ANC service utilization in Ethiopia, both related to the health system and community level. Furthermore, the review highlighted potential research areas related to health systems and community-level determinants in Ethiopia that require critical analysis and additional research.

### The specific objectives of the scoping review are:

- To investigate the current state of comprehensive knowledge regarding national ANC utilization.
- To identify the barriers influencing the utilization of ANC services.
- To determine recent knowledge gaps and highlight potential research areas in the ANC service of Ethiopia.

## Methods

### Design

A scoping review assessed the utilization and determinants of ANC service in Ethiopia. The Arksey and O’Malley five-step scoping review framework was used as an organizing principle for the review process. It consists of the following steps: i) defining the research question, ii) finding pertinent studies, iii) choosing studies and reports, iv) charting the data, and v) compiling, summarizing, and reporting the results [25]. The Preferred Reporting Items for Systematic Reviews and Meta-Analyses extension for Scoping Reviews (PRISMA-ScR) Checklist is utilized when presenting the results of this review [26]. The checklist is provided as S1 File.

### Search strategy

The Joanna Briggs Institute (JBI) PCC (Population/Concept/Context) framework [27] was utilized to present the search strategy and criteria for inclusion and exclusion.

All studies and reports were systematically searched from national and international databases utilizing Boolean operators with search terms such as antenatal care, maternal health care, prenatal care, utilization, predictors, determinants, associated factors, barriers, facilitators, women of reproductive age, and Ethiopia. PubMed, Medline, WHO Library, CINAHL, EMBASE, Science Direct, HINARI, Cochrane or Wiley Library, and Google Scholar were searched to obtain studies conducted in Ethiopia between 2002 and 2024. As supporting information, more details of the PubMed, Medline, CINAHL, and EMBASE search strategies are given (S2 File). Grey literature was found by looking through the first ten pages of the Google search results. Furthermore, the websites of the Multiple Indicator Cluster Surveys (MICS), the Reproductive Health Survey (RHS) database, the Demographic and Health Surveys (DHS) database, and the United States Agency for International Development (USAID) publications were accessed. To ensure that no pertinent articles were missed during the search, the reference lists of the retrieved studies were examined. Furthermore, this review included unpublished papers, guidelines, and reports from the Ethiopian Federal Ministry of Health (FMOH). On October 1, 2024, the literature search was completed.

### Selection criteria to include studies

The studies in this scoping review were selected using the following inclusion criteria:

**Study design**: all studies reported ANC service utilization in Ethiopia.

**Study setting**: both community- and institution-based studies in Ethiopia.

**Study period**: studies conducted from September 2002 to September 2024. 2002 was chosen as the starting point since the Ethiopian government launched focused ANC during this time.

**Outcome**: a pregnant woman with at least one ANC visit in Ethiopia.

**Language**: a study accessible with only full text in English.

**Publication status**: both unpublished and published studies.

**Administrative reports and national assessments** that emphasized the utilization and barriers of ANC service in Ethiopia.

**Publications** that do not include comprehensive methods and results (for example, conference abstracts, commentary, and study protocols) were excluded.

**Ethics statement:** Not applicable

### Data abstraction and analysis

Citations were filled in into Rayyan, a web-based application designed to help screen studies and data extraction procedures [28]. A standard data abstraction spreadsheet extracted data from the included studies. Two authors (AY and FGG) were involved in data abstraction format development to ensure that the tool accurately captured all needed data to respond to the review queries. Two authors independently conducted data abstraction from the included studies. From each included study, the authors collected the name of the first author(s), years of publication, data collection period, the proportion of ANC service use, determinants, study settings, and design. Cohen’s Kappa was used to calculate the level of agreement among the two authors. Disagreements between the two authors were resolved through discussion and consultation with a third party for any ongoing disagreements. We utilized a descriptive synthesis technique to analyze the data. The analytical discussion centered on reviewing and summarizing ANC utilization coverage, determinants, and barriers to ANC utilization that require more focused attention.

## Results

### Description of search results and features of the included studies

**Fig 1:**
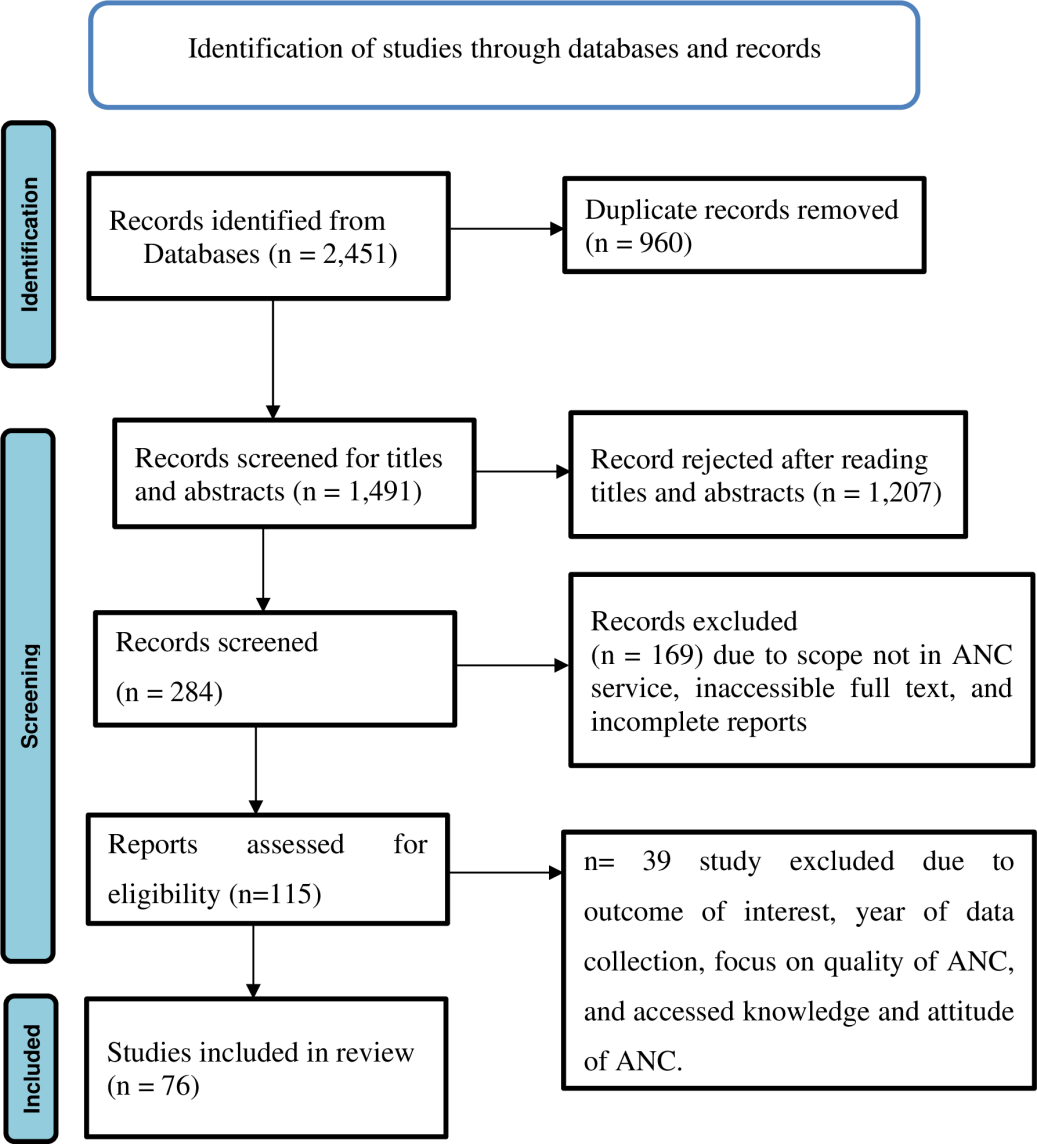
Review profile.

Initially, the bibliographic database searches yielded 2350 titles and abstracts. An additional 101 titles and abstracts were discovered during the grey literature search. After removing duplicates, the 1491 titles and abstracts were screened, yielding 1207 records rejected after reading titles and abstracts and 284 potential records for inclusion. After full-text screening, the review included 76 records (62 peer-reviewed articles and 14 grey literature documents). Fig 1 [29] depicts a flowchart based on PRISMA guidelines.

This review included 76 studies and national reports about Ethiopian maternal health programs (Fig 1). Most of the studies were cross-sectional in design and were published. Two national maternal health service coverage surveys and four EDHS studies were among the included reports. Since 2000, the others have been conducted in various parts of the country. National documents and unpublished administrative reports from the Health Management Information System (HMIS) were also included for review.

### Main findings

The following themes were used to summarize the findings: ANC service coverage and timeliness of its initiation, dropout rate, determinants of ANC service utilization, health service availability, community involvement, and gender role.

### ANC service utilization

Overall, at least one ANC utilization increased from 28% in 2005 to 74%, whereas four or more ANC service utilization increased from 12% in 2005 to 43% in 2019 in Ethiopia. A significant proportion (31%) of pregnant women dropped out of ANC utilization, and only 28% attended timely initiation of ANC service (Table 1).

**Table 1:** The Ethiopian national evidence on ANC utilization coverage and timeliness.

S.n	Author	Design	Results	Conclusions
1	FMO, 2002	Cross-sectional	ANC once = 26% ANC + 4 = 11% ANC use in first 3 months = 5%	Significant variations in the coverage between regions High urban and rural differences and dropouts
2	CSA, 2005	Cross-sectional	ANC once = 28% ANC + 4 = 12% ANC use in first 3 months = 6%	Significant variations in the coverage between regions High urban and rural differences and dropouts
3	Worku et al., 2022	Cross-sectional	ANC once = 93% ANC + 4 = 54% ANC use in first 4 months = 61%	High progress but not uniform in all districts of the state Significant dropout rate
4	CSA, 2011	Cross-sectional	ANC once = 34% ANC + 4 = 19% ANC use in first 4 months = 11%	Inequality between regions persisted with a significant dropout rate
5	EPHI, 2022	Cross-sectional	ANC once = 79% ANC + 4 = 49% ANC use in first 4 months = 32%	Access and utilization are increased in most regions Regional, urban, and rural disparities slightly closing
6	FMOH, 2014	HMIS	ANC once = 97% Somali region = 41.6% Tigray, Oromia, SNNP, Harari, and Dire Dawa = 100%	Achievement above target bust significant regional variation
7	CSA, 2016	Cross-sectional	ANC once = 62% ANC + 4 = 32% ANC use in first 4 months = 20%	The EDHS surveys report showed a slow progress in ANC coverage
8	CSA, 2019	Cross-sectional	ANC once = 74 ANC + 4 = 43% ANC use in first 4 months = 28%	Showed progress with a significant dropout rate and great regional, urban, and rural disparity
8	FMOH,2014	HMIS	ANC + 4 = 70% Afar region = 46% Addis Ababa = 100%	Showed good progress since 2010 coverage of 86%
9	UNICEF, 2022	Estimates	ANC once = 84 ANC + 4 = 68% ANC use in first 4 months = 38%	Revealed improvement from previous estimates but a vast disparity between regio
10	FMO, 2018	HMIS	ANC once = 99%	Above target but significant regional variation

### Determinants of antenatal care service utilization

This review identified the main determinants of ANC utilization in Ethiopia, such as socio-economic and demographic, socio-cultural, knowledge of mothers regarding the ODS and practices of women about BPCR, maternal decision-making authority, and gender role, perceived quality of ANC, health facility, and obstetric-related determinants (Table 2).

**Table 2:** Main identified determinants of antenatal care service utilization in Ethiopia.

s.no	Main determinants	Sub-categories
1	Socio-economic and demographic determinants	Maternal age Educational status of women and their husbands Occupational status of women and their husbands Place of residence Household wealth index or status Marital status Exposure to media and information
2	Socio-cultural determinants	Availability of traditional healer Availability of spiritual and religious healer Cultural belief
3	Knowledge of mothers regarding the ODS and practices of women regarding BPCR	Awareness Knowledge Attitude Practice
4	Maternal decision-making authority and gender role	Autonomy of women Women’s role in household
5	Health facility-related determinants and perceived quality of ANC	Cost of services Availability and accessibility of service Availability of medical supplies, drugs, and important equipment Attitude or communication of health care provider Bad history and experience with health facilities and systems Supposed quality of care
6	Obstetric related determinants	Gravidity and parity Abortions and stillbirths Unplanned pregnancy Maternal age at marriage and first pregnancy Experience of ODS Place of delivery of the elder child Birth spacing

## Discussion

### ANC service utilization coverage

Seven national surveys were conducted to evaluate ANC service coverage at the national level: two national maternal health care coverage surveys [30,31] and five EDHS studies [18,32–35]. Furthermore, three WHO/United Nations International Children’s Emergency Fund (UNICEF) reports [19,20,36] and three FMOH administrative reports [21,37,38] were reviewed. According to these studies and administrative reports, Ethiopia’s ANC service utilization coverage has been rising recently. Based on the most recent mini-EDHS 2019 report, the country’s at least ANC utilization coverage has increased from 28% in 2005 to 74%, whereas four or more ANC service utilization increased from 12% in 2005 to 43% in 2019 [18]. However, there have been persistent regional differences in Ethiopia since 2005, with pastoralist regions consistently having very low ANC utilization rates. The ANC service utilization coverage rate varies significantly between urban and rural areas over time. The EDHS surveys’ full completion of recommended ANC visits was significantly lower than administrative reports, ANC coverage survey findings, and UNICEF/WHO estimates [18,20,21,36]. Overall, there was low access to and utilization of ANC services, according to the review’s findings. The Afar and Somalia regions had the least access to and utilization of ANC services, with the highest in Addis Ababa [18,21,31,36]. The trend in ANC service coverage also indicated that ANC coverage is far below the national target [18] (Table 1).

Additionally, 47 pocket studies were conducted to determine the ANC service utilization coverage in various areas of the nation, and utilization ranged between 27% in the Somali region [39] and 90.6% in the Oromia region [40]. Of them, 13 studies were conducted in the southern regions, where the ANC service utilization coverage ranged from 28.5 to 87.6% [41,42]. Eleven studies from the Amhara region also reported overall ANC utilization between 32.3–63.0% [43,44]. Further, nine studies from the Oromia regional state reported variable coverage of ANC service utilization across the region, with the lowest at 37.4% [45] and the highest at 90.6% [40], whereas five studies from the Tigray region reported utilization coverage between 54 and 76% [46,47]. Finally, studies from Afar, Somalia, and Benishangul Gumuz reported that utilization of ANC was 42.4%, 27–66%, and 36.1–66.1%, respectively (See S2 File). Most of the studies’ findings generally indicated that ANC service utilization coverage was low and that progress was uneven throughout the country’s region. Variations in the study period, design, sample size, and methodology of the sample selection, along with disparities in the information source, health service availability and accessibility, health facility accessibility, level of socio-economic development, and health care providers to population density, may account for variations in the coverage.

### Antenatal care dropout rate

The national surveys, WHO/UNICEF estimates, and FMOH administrative reports indicated that the ANC dropout rate was unacceptable and very high at the country level [19–21,30,37]. According to a recent EDHS report, the ANC dropout rate was not within an acceptable range (31%). This dropout rate is significantly higher than the target set under the health sector transformation plan for 2025 [13]. Similarly, in every small-pocket study reviewed, ANC 4 coverage was significantly lower than ANC 1 coverage, with unacceptable ranges in dropout rates leading to a more significant proportion of women who received only partial ANC services [48–51]. The proportion of non-utilized women differed between study areas, with the Somali region significantly declining [39]. The survey results were generally less than the national estimates and administrative reports [13,19–21,38]. These findings indicate that Ethiopian women are not getting the recommended ANC services per the WHO recommendations.

### Timely initiation of the first antenatal care visit

Timely initiation of the first antenatal care contact is defined as women who have their first visit within the first trimester of their pregnancy as per WHO recommendations on ANC for a positive pregnancy experience [16]. Evidence from the national surveys, WHO/UNICEF estimates, and MOH administrative reports indicated that the delayed initiation of ANC was very high, with high regional, urban, and rural disparities [19–21,30,37]. From 2005 to 2019, the percentage of women who had not received their first ANC visits during the recommended time (first trimester) remained consistently low and stable (11 vs. 28%) [18].

Timely initiation of ANC service was also assessed in 15 local studies, which showed that the early initiation of the first ANC visit ranged between 27.5 [52] and 58% [53] and had a much better figure as compared with the early initiation of ANC coverage reported by national surveys [18]. The evidence also indicated that early initiation of ANC services is not considered in the national maternal and child health program (Table 1).

### Determinants of antenatal care service utilization

Barriers and facilitators of ANC service utilization were mainly tied to service access, appropriateness, acceptability, the health system, and health facility constraints. The main determinants linked to disparities in coverage of ANC service utilization are complex and include place of residence, region, service access, media accessibility, distance from a health facility, and individual socio-demographic characteristics, which were found to be predictive of ANC service utilization based on the evidence currently available. The results of seven national surveys and 51 local studies on ANC service uptake barriers and facilitators are presented here. The findings from different works of literature were presented as follows.

### Socio-economic and demographic determinants

Several socio-economic and demographic determinants of the individual influence the essential use of ANC. For instance, women’s age, the levels of education of women and their spouses, place of residence, occupation, use of media, information about MHC, and household wealth index have been frequently studied as determinants of ANC [54–62]. We will discuss each in detail below using evidence from different kinds of literature.

### Maternal age

Previous studies reported controversial findings on the relationship between maternal age and ANC. Numerous observational studies were conducted in Rural India [63], Wollega Zone [64], Tigray region [65], Munisa Woreda [66], and Kilite-Awlaelo Health and Demographic Surveillance System in the Tigray region of Ethiopian [67] reported an inverse relationship. Conversely, the studies conducted in Ethiopia reported a positive association [68–70]. Therefore, this variable requires meta-analysis to fix paradox findings from individual studies, but we have not abstracted data from individual studies due to the nature of scoping reviews.

### Educational status of women and their husbands

Different studies reported that the educational status of women and their husbands is one of the key components in increasing the utilization of ANC and is considered one of the single important determinants associated with obtaining ANC [63–67,71–75]. In Ethiopia, 10 studies from different parts of Ethiopia and three systematic reviews and meta-analysis studies also confirmed that the education status of women was significantly associated with ANC [64,70–72,74,75]. In general, all studies assessing the association of women’s educational status with ANC agree that the odds of ANC increased with women’s education status. The earlier studies reported that educated mothers have better exposure/access to health information regarding modern health care, better capability to communicate with HCPs, awareness, and knowledge to overcome traditional and cultural barriers, and increased decision-making power. Also, a higher educational level authorizes mothers to control healthcare resources and increase access to quality maternal health services. Moreover, education raises women’s incomes, improving their capability to offer financial help to their families and consequently contributing to household decision-making decisions, including decisions on fiscal expenditure on their own and child health care.

### Occupational status of women and their husbands

Different studies reported that women and their husbands’ occupation status is one of the key components in increasing utilization of ANC [65,67,73,74]. For instance, a community-based study from the Tigray region, Ethiopia, showed that husbands´ occupation was significantly associated with maternal health service, particularly ANC [65].

### Place of residence

In several studies, researchers found that the place of residence influences the ANC. All of these studies reported a significant positive relationship between utilization of ANC and urban residence [64,65,67,72–74]. Similarly, the 2019 mini DHS of Ethiopia reported that the utilization of ANC increased in urban settings compared to rural settings [55].

### Household wealth index or status

In general, mothers in the highest wealth index groups tend to reveal patterns of higher utilization of ANC than mothers in the lowest wealth index groups [70,71,74]. Similarly, the mini EDHS 2019 showed that the utilization of MHS increased with increased household wealth index or status [55].

### Marital status

Many studies conducted in different parts of Ethiopia reported that the utilization of ANC in married women is larger as compared to divorced, single, and widowed women [62,65,76]. On the other hand, a study finding from the Tigray region, Ethiopia, reported that ANC utilization is higher in divorced women as compared to widowed and single women, which is a unique result [65].

### Exposure to media and information

In several studies, researchers reported that the ANC is affected by the availability of information and exposure to media regarding the ANC [72,73,75].

### Socio-cultural determinants

The other vital determinant of ANC, particularly in Africa, is the socio-cultural background of mothers. The socio-cultural view is an important determinant of ANC utilization, and many studies have suggested that medical need is determined not only by the presence of illness but also by the socio-cultural perspective of disease [77–79]. For instance, a qualitative study in Ethiopia showed that mothers and their families only seek ANC during the antepartum period for obstetric complications if prayer and herbal or local medicines are conquered [79].

### Knowledge of mothers regarding the ODS and practices of women regarding BPCR

The individual-level determinants that influence the use of ANC are lack of knowledge of the benefit of maternal care and any potential complications of gestation and delivery, the experience of ODS, and the practices of women about BPCR [73,75,80]. Studies conducted in different places reported that maternal individual knowledge regarding the ODS and the skill of BPCR were important determinants for the utilization of ANC [81–84]. Several previous studies reported that the major determinants for not utilizing ANC were maternal unawareness of the benefits of ANC, lack of enough information regarding the benefits of ANC, and dissatisfaction with earlier ANC [62,65,76].

### Maternal decision-making authority and gender role

Women’s decision-making authority is one of the most significant determinants that affect ANC [63,64]. Men are responsible for making decisions and controlling all resources in most households for cultural reasons. Also, they make decisions about where and when mothers should obtain ANC. Therefore, the low status of mothers and their gender role in the community influences them to identify their problems regarding health demands [85]. A study in Ethiopia reported that around 5% of mothers who did not follow ANC at all provided their husband‘s disagreement as a descriptive reason for non-utilizing the ANC service [86].

The decision to seek health care is a significant determinant of the utilization of MHS. However, the practice is still unsatisfactory, particularly in Ethiopia. A community-based study in Southern Ethiopia indicated that women’s autonomy to decide on health care was an independent determinant of ANC [87]. Similarly, the study from the West Shewa zone of the Oromia regional state of Ethiopia also reported that the autonomous decision-making power of women was a strong determinant of ANC [88]. Furthermore, several studies conducted in Ethiopia to assess ANC reported that maternal autonomy in making decisions regarding household issues was a significant determinant of ANC utilization [23,64,76].

### Health facility-related determinants and perceived quality of ANC

Health facility related-determinants that influence ANC are the cost of services, availability, and accessibility of service, availability of medical supplies, drugs, and important equipment, attitude or communication of health care provider, bad history and experience with health facilities and systems, and supposed quality of care [23,56–59,61,62,89]. Health institutions’ physical coverage remains a considerable challenge, particularly in rural settings. In most rural settings, 1 in 3 mothers is greater than 5 km from the nearby health institution [90]. The lack of proper public transport and road infrastructure makes service access challenging, particularly during obstetric complications. Also, walking is the principal method of transport, even used for mothers in labor. Due to these reasons, poor mothers will obtain ANC from less-skilled healthcare providers who are considerably accessible in most remote settings [91–93].

The quality of ANC, which is perceived as quality care, plays a significant role in the decision to obtain care. It is also associated with an individual’s evaluation of service provision, which highly depends on their own experiences with the health care system and that individual knows [90]. Several studies conducted in developed and developing countries reported that maternal perception of the quality of ANC significantly influenced maternal utilization of existing services during pregnancy [70,73,87]. Furthermore, different studies reported that distance from the health organization, lack of health insurance, ability to afford health care service at the health facility, lack of companionate and respectful care in the health facility, low skills of health care providers, information about maternal health care, and traveling time that takes to reach to health institution are significant determinants of ANC [65,70,87].

### Obstetric related determinants

The obstetric determinants such as gravidity, parity, abortions, stillbirths, unplanned pregnancy, maternal age at marriage and first pregnancy, knowledge of ODS, the experience of ODS, place of delivery of the elder child, and birth spacing were significant determinants of ANC [70,72,81–84]. A Debre Tabor Town, Ethiopia study indicated that planned pregnancy was an important determinant for focused ANC service utilization [76]. Similarly, a systematic review and meta-analysis study from Ethiopia also reported that planned pregnancy was significantly associated with ANC [64].

### The identified priority areas for research in the antenatal care service

Based on a comprehensive review of the existing body of knowledge and expert reviews using the Delphi method, the following top-priority areas for future studies have been identified:

- Strengthening the linkage of community-based outreach services
- Adoption and adaptation of new technologies for the antenatal care service
- Availability of supplies, equipment, and drugs at the health facility level
- A comprehensive community-level data confirmation mechanism for the antenatal care service
- Active community engagement and healthcare provider-client communication
- Effects of electronic community health information system implementation on antenatal care services
- Women’s autonomy and empowerment in antenatal care services
- Antenatal care service provision mechanisms in displaced communities
- Revitalizing antenatal care services in pastoralist communities and slum urban settings
- Mechanisms that increase husband involvement

## Conclusions

The available evidence indicates that the national ANC service coverage is below the national target, even though the proportion of women who have received recommended ANC visits is rising over time. The timely initiation of ANC visits is much lower than the total ANC service coverage. Survey findings, administrative reports, and global estimates show large disparities in ANC utilization between regions and urban/rural settings. The evidence also showed significant disparities in ANC service coverage based on socioeconomic status. Additionally, there is inadequate community involvement in the ANC service. The factors influencing ANC service coverage vary by context, necessitating the development and implementation of targeted interventions. Nationwide, there is a dearth of implementation science evidence. Concerning ANC service barriers, there is sufficient evidence on individual-level determinants, but evidence on community-level and health system-level determinants is limited. Additional research priorities have been identified, and it is necessary to investigate any remaining utilization barriers for the ANC service while focusing more on the research priorities that have been identified.

Therefore, to increase women’s income, the government and stakeholders should implement economic reforms specifically targeting women. These reforms should include encouraging women to participate in rural savings and credit cooperative organizations, promoting ANC in the media, encouraging women to enroll in and complete their education, supporting women’s participation in household decision-making, and expanding the mechanism of ANC service messages to reach the largest proportion of women of reproductive age groups. Any program should promote BPCR practice, raise community understanding of the advantages of ANC utilization, and teach them how to overcome sociocultural views that prevent them from using ANC services.

## Supporting information

S1 File Preferred Reporting Items for Systematic reviews and Meta-Analyses extension for Scoping Reviews (PRISMA-ScR) Checklist. (PDF)

S2 File Search strategy. (DOCX)
