# Supplementary material for: What we know and don’t know about the antenatal care service utilization in Ethiopia: A scoping review of the literature
Source: PLoS One. 2025 May 30;20(5):e0321882. doi: 10.1371/journal.pone.0321882 (PMC12124521; doi:10.1371/journal.pone.0321882)
Supplement: S2 File — (DOCX) [file pone.0321882.s002.docx]

**Supplementary file 2:** Search strategy

Search: **(((((((((Antenatal care) OR (Maternal health care)) OR (Prenatal care)) AND (Utilization)) OR (Utilisation)) AND (Predictors)) OR (Determinants)) OR (Associated factors)) OR (barriers)) OR (facilitators)) AND (Women of reproductive age)) AND (Ethiopia)**

((((("prenatal care"[MeSH Terms] OR ("prenatal"[All Fields] AND "care"[All Fields]) OR "prenatal care"[All Fields] OR ("antenatal"[All Fields] AND "care"[All Fields]) OR "antenatal care"[All Fields] OR ("maternal health services"[MeSH Terms] OR ("maternal"[All Fields] AND "health"[All Fields] AND "services"[All Fields]) OR "maternal health services"[All Fields] OR ("maternal"[All Fields] AND "health"[All Fields] AND "care"[All Fields]) OR "maternal health care"[All Fields]) OR ("prenatal care"[MeSH Terms] OR ("prenatal"[All Fields] AND "care"[All Fields]) OR "prenatal care"[All Fields])) AND ("statistics and numerical data"[MeSH Subheading] OR ("statistics"[All Fields] AND "numerical"[All Fields] AND "data"[All Fields]) OR "statistics and numerical data"[All Fields] OR "utilization"[All Fields] OR "utilisation"[All Fields] OR "utilisations"[All Fields] OR "utilise"[All Fields] OR "utilised"[All Fields] OR "utilises"[All Fields] OR "utilising"[All Fields] OR "utilities"[All Fields] OR "utility"[All Fields] OR "utilizations"[All Fields] OR "utilize"[All Fields] OR "utilized"[All Fields] OR "utilizer"[All Fields] OR "utilizers"[All Fields] OR "utilizes"[All Fields] OR "utilizing"[All Fields])) OR ("statistics and numerical data"[MeSH Subheading] OR ("statistics"[All Fields] AND "numerical"[All Fields] AND "data"[All Fields]) OR "statistics and numerical data"[All Fields] OR "utilization"[All Fields] OR "utilisation"[All Fields] OR "utilisations"[All Fields] OR "utilise"[All Fields] OR "utilised"[All Fields] OR "utilises"[All Fields] OR "utilising"[All Fields] OR "utilities"[All Fields] OR "utility"[All Fields] OR "utilizations"[All Fields] OR "utilize"[All Fields] OR "utilized"[All Fields] OR "utilizer"[All Fields] OR "utilizers"[All Fields] OR "utilizes"[All Fields] OR "utilizing"[All Fields])) AND ("predictor"[All Fields] OR "predictors"[All Fields])) OR ("analysis"[MeSH Subheading] OR "analysis"[All Fields] OR "determination"[All Fields] OR "determinant"[All Fields] OR "determinants"[All Fields] OR "determinate"[All Fields] OR "determinated"[All Fields] OR "determinates"[All Fields] OR "determinating"[All Fields] OR "determinations"[All Fields] OR "determine"[All Fields] OR "determined"[All Fields] OR "determines"[All Fields] OR "determining"[All Fields]) OR (("associate"[All Fields] OR "associated"[All Fields] OR "associates"[All Fields] OR "associating"[All Fields] OR "association"[MeSH Terms] OR "association"[All Fields] OR "associations"[All Fields]) AND ("factor"[All Fields] OR "factor s"[All Fields] OR "factors"[All Fields]))) AND (("womans"[All Fields] OR "women"[MeSH Terms] OR "women"[All Fields] OR "woman"[All Fields] OR "women s"[All Fields] OR "womens"[All Fields]) AND ("reproduction"[MeSH Terms] OR "reproduction"[All Fields] OR "reproductions"[All Fields] OR "reproductive"[All Fields] OR "reproductively"[All Fields] OR "reproductives"[All Fields] OR "reproductivity"[All Fields]) AND ("age"[Journal] OR "age omaha"[Journal] OR "age dordr"[Journal] OR "adv genet eng"[Journal] OR "age"[All Fields])) AND ("ethiopia"[MeSH Terms] OR "ethiopia"[All Fields] OR "ethiopia s"[All Fields])

**Supplementary file 2 Table 1:** The systematic PubMed search method and limited by utilizing English language and studies done between 2002 to 2024 years.

| Search # | Query | Results | Time |
| --- | --- | --- | --- |
| 11 | (((((((((#1) OR (#2)) OR (#3)) AND (#4)) OR (#5)) AND (#6)) OR (#7)) OR (#8)) AND (#9)) AND (#10) | **1,011** | 15:01:47 |
| 10 | Ethiopia | 30,825 | 14:59:39 |
| 9 | Women of reproductive age | 112,473 | 14:59:09 |
| 8 | Associated factors | 1,860,911 | 14:58:43 |
| 7 | Determinants | ######## | 14:58:26 |
| 6 | Predictors | 450,350 | 14:58:04 |
| 5 | Utilisation | 4,048,246 | 14:57:37 |
| 4 | Utilization | 4,048,246 | 14:57:21 |
| 3 | Prenatal care | 54,227 | 14:56:53 |
| 2 | Maternal health care | 105,643 | 14:56:20 |
| 1 | Antenatal care | 65,564 | 14:55:23 |

**Supplementary file 2 Table 2:** The systematic Medline search method and limited by utilizing English language and studies done between 2002 to 2024 years.

| Search # | Searches | Results | Search type | Actions |
| --- | --- | --- | --- | --- |
| 1 | Antenatal care | 6288 | Advanced | Display |
| 2 | Maternal health care | 12723 |  |  |
| 3 | Prenatal care | 21534 |  |  |
| 4 | 1 or 2 or 3 | 39652 |  |  |
| 5 | Utilization | 15672 |  |  |
| 6 | Determinants | 14356 |  |  |
| 7 | Associated factors | 33564 |  |  |
| 8 | 4 and 5 | 1547 |  |  |
| 9 | 4 or 6 | 162895 |  |  |
| 10 | 4 and 7 | 298 |  |  |
| 11 | Ethiopia | 9569 |  |  |
| 12 | 8 or 9 | 164987 |  |  |
| 13 | 9 and 10 | 281 |  |  |
| 14 | 12 and 13 | 243 |  |  |
| 15 | 4 or 5 or 6 or 7 | 33678 |  |  |
| 16 | 11 and 15 | 812 |  |  |
| 17 | 8 or 9 or 10 | 162145 |  |  |
| 18 | 11 and 17 | 612 |  |  |
| 19 | 4 and 11 | 330 |  |  |
| 20 | 18 or 19 | 619 |  |  |
| 21 | 18 and 19 | 512 |  |  |
| 22 | 20 and 21 | **512** |  |  |

**Supplementary file 2 Table 3:** The systematic CINAHL search method and limited by utilizing English language, age and studies done between 2002 to 2024 years.

| **Search ID#** | **Search Terms** | **Search Options** | **Last Run Via** | **Results** |
| --- | --- | --- | --- | --- |
| S7 | S1 AND S3 AND S4 AND S65 | Search modes - Boolean/Phrase | Interface - EBSCOhost Research Databases  Search Screen - Advanced Search  Database - CINAHL Complete | **211** |
| S6 | S1 AND S3 AND S4 AND S5 | Search modes - Boolean/Phrase | Interface - EBSCOhost Research Databases  Search Screen - Advanced Search  Database - CINAHL Complete | 211 |
| S5 | Ethiopia | Search modes - Boolean/Phrase | Interface - EBSCOhost Research Databases  Search Screen - Advanced Search  Database - CINAHL Complete | 3,557 |
| S4 | Associated factors OR Factors associated OR Influencing factors OR Predictors OR Predicting factors OR ( Correlates or determinants ) OR ( Determinants or factors ) OR ( risk factors or protective factors ) | Search modes - Boolean/Phrase | Interface - EBSCOhost Research Databases  Search Screen - Advanced Search  Database - CINAHL Complete | 1,155,068 |
| S3 | S1 OR S2 | Search modes - Boolean/Phrase | Interface - EBSCOhost Research Databases  Search Screen - Advanced Search  Database - CINAHL Complete | 29,145 |
| S2 | Utilization OR utilisation | Search modes - Boolean/Phrase | Interface - EBSCOhost Research Databases  Search Screen - Advanced Search  Database - CINAHL Complete | 13,568 |
| S1 | (antenatal care or prenatal care ) OR First antenatal care OR Antepartum care OR ANC OR PNC OR Antenatal follow up OR Antenatal period OR Antenatal service OR Antenatal care visit OR Antenatal presentation OR Antenatal attendance | Search modes - Boolean/Phrase | Interface - EBSCOhost Research Databases  Search Screen - Advanced Search  Database - CINAHL Complete | 298,676 |

**Supplementary file 2 Table 4:** The systematic EMBASE search method and limited by utilizing English language and studies done between 2002 to 2024 years.

| Search # | Searches | Results |
| --- | --- | --- |
| 1 | Antenatal care | 132377 |
| 2 | Maternal health care | 12723 |
| 3 | Prenatal care | 21534 |
| 4 | First antenatal care | 705334 |
| 5 | Antepartum care | 342066 |
| 6 | Antenatal follow up | 485524 |
| 7 | Antenatal period | 304064 |
| 8 | Antenatal service | 311668 |
| 9 | Antenatal care visit | 131988 |
| 10 | Antenatal presentation | 28661 |
| 11 | Antenatal attendance | 795139 |
| 12 | 1 or 2 or 3 or 4 or 5 or 6 or 7 or 8 or 9 or 10 or 11 | 2244549 |
| 13 | Utilization | 134573 |
| 14 | Utilization | 133994 |
| 15 | 13 or 14 | 2867897 |
| 16 | Determinants | 154332 |
| 17 | Associated factors | 117675 |
| 18 | Influencing factors | 7930 |
| 19 | Predicting factors | 261656 |
| 20 | Correlates | 182554 |
| 21 | Risk factors | 962833 |
| 22 | 16 or 17 or 18 or 19 or 20 or 21 | 2073703 |
| 23 | Ethiopia | 13770 |
| 24 | 12 and 15 and 22 and 23 | **1123** |
